# Supplementary material for: Outcomes and Prognostic Factors in Critical Patients with Hematologic Malignancies
Source: J Clin Med. 2023 Jan 26;12(3):958. doi: 10.3390/jcm12030958 (PMC9918099; doi:10.3390/jcm12030958)
Supplement: Supplementary file 1 [file jcm-12-00958-s001.zip › jcm-2151346-supplementary.pdf]

Supplementary Table S1. Comparison of the severity of illness scores and mortality of the present study to the previous literature.

	SOFA Score	SAPS II	APACHE II Score	ICU Mortality	Hospital Mortality
Lloyd-Thomas et al., 1988 (10)			20.2 (12–26) in hospital survivors 30.6 (14–48) in hospital deaths	63.3%	78.3%
Massion et al., 2002 (14)		54 (40–70)	24 (17–28)	38.1%	60.7%
Benoit et al., 2003 (11)		53 (17.8)	26 (7.7)	41.9%	54%
Hampshire et al., 2009 (9)			24.4 (7.9)	43.1%	59.2%
Bird et al., 2012 (24)			21 (16–25)	33.7%	45.7%
Al-Zubaidi et al., 2018 (15)	7.6 (3.7)	48.3 (14.9)	23.4 (7.3)	24.8%	45.3%
Present study, 2022	11 (9–15)	63 (51.5–77)	28 (23–34)	55.9%	71.8%

Supplementary Table S2. Cause of death in hospital.

	N = 153
Septic shock	94 (61.4%)
Respiratory failure	31 (20.3%)
Neurological disorder	10 (6.5%)
Intracerebral hemorrhage	4 (2.6%)
Subarachnoid hemorrhage	1 (0.7%)
Subdural hematoma	1 (0.7%)
Large infarction	1 (0.7%)
Brain swelling	2 (1.3%)
Post CPR hypoxic encephalopathy	1 (0.7%)
Liver failure	6 (3.9%)
Non-CNS bleeding	6 (3.9%)
Gastrointestinal bleeding	5 (3.3%)
Pulmonary hemorrhage	1 (0.7%)
Renal failure	5 (3.3%)
Heart failure	1 (0.7%)

Abbreviations: CNS = central nervous system; CPR = cardiopulmonary resuscitation

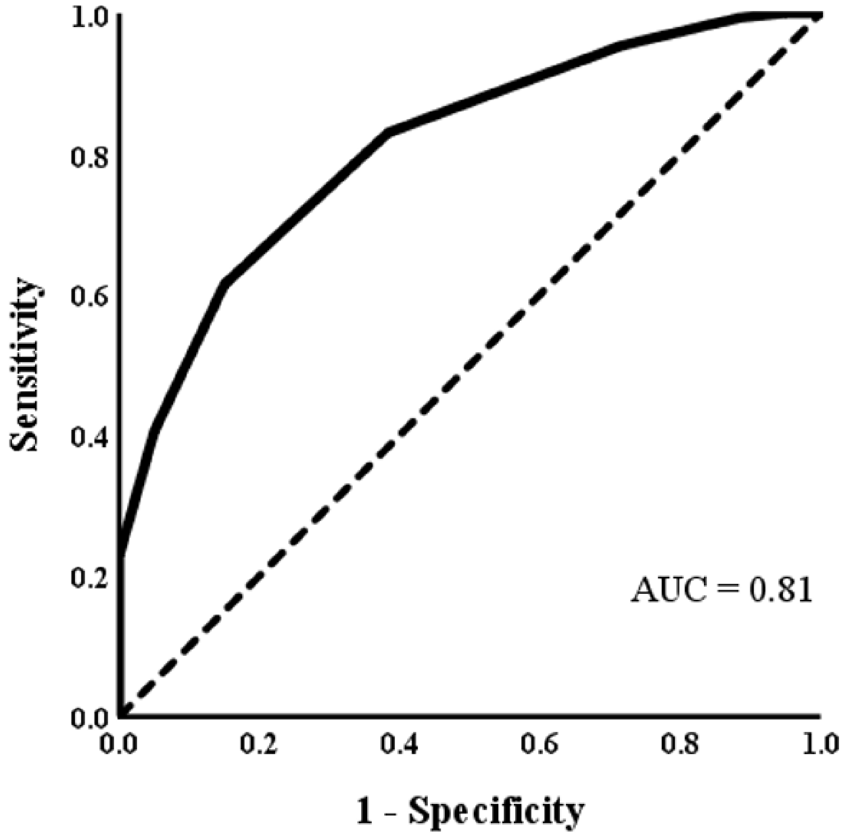

Supplementary Figure S1. ROC curve for predicting hospital mortality in patients with HMs admitted to the ICU based on the HHM score.

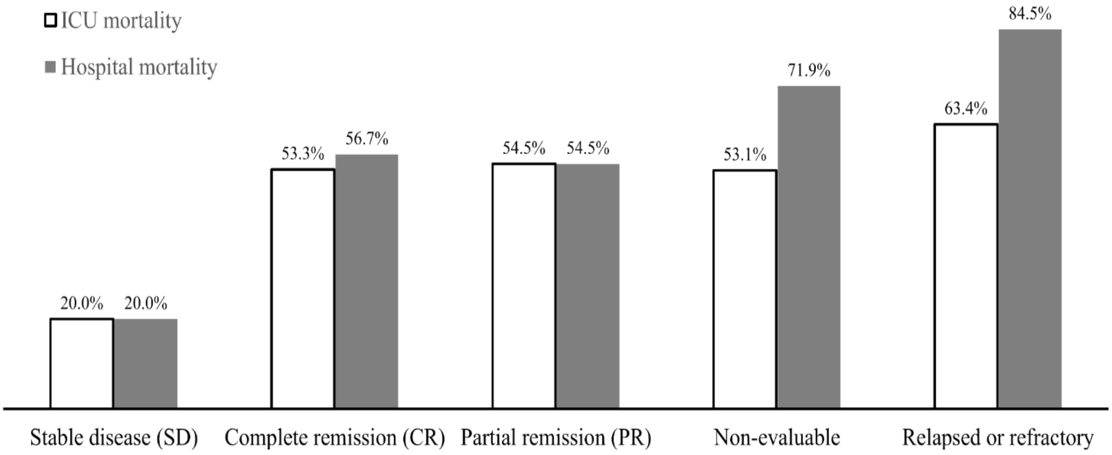

Supplementary Figure S2. ICU and hospital mortality among patients with different disease statuses.

Supplementary Table S3. Details of ICU-acquired infections.

	**N = 82**
Source of infection	
Pneumonia	52 (63.4%)
Blood stream infection	50 (61.0%)
Urinary tract infection	4 (4.9%)
Multidrug-resistant organism	49 (59.8%)
Multiple pathogen identification	30 (36.6%)
Candidemia	7 (8.5%)
Pathogens	
Gram-positive organisms	
Enterococcus faecium	16 (19.5%)
Corynebacterium spp.	5 (6.1%)
Staphylococcus aureus	3 (3.7%)
Other gram-positive organisms	4 (4.9%)
Gram-negative organisms	
Acinetobacter baumannii/calcoaceticus complex	22 (26.8%)
Stenotrophomonas maltophilia	14 (17.1%)
Klebsiella pneumoniae	11 (13.4%)
Pseudomonas aeruginosa	8 (9.8%)
Acinetobacter spp.	7 (8.5%)
Elizabethkingia spp.	4 (4.9%)
Burkholderia spp.	3 (3.7%)
Escherichia coli	2 (2.4%)
Proteus mirabilis	2 (2.4%)
Other gram-negative organisms	7 (8.5%)
Fungus	
Candida glabrata	4 (4.9%)
Candida albicans	3 (3.7%)
Candida tropicalis	3 (3.7%)
Other fungi	4 (4.9%)
